# The Swi3 protein plays a unique role in regulating respiration in eukaryotes

**DOI:** 10.1042/BSR20160083

**Published:** 2016-06-30

**Authors:** Sneha Lal, Md Maksudul Alam, Jagmohan Hooda, Ajit Shah, Thai M. Cao, Zhenyu Xuan, Li Zhang

**Affiliations:** * Department of Biological Sciences, Center for Systems Biology, University of Texas at Dallas, Mail Stop RL11, 800 W. Campbell Road, Richardson, TX 75080, U.S.A.

**Keywords:** aerobic respiration, BAF155, BAF170, bioenergetics, oxygen consumption, Swi3

## Abstract

Recent experimental evidence increasingly shows that the dysregulation of cellular bioenergetics is associated with a wide array of common human diseases, including cancer, neurological diseases and diabetes. Respiration provides a vital source of cellular energy for most eukaryotic cells, particularly high energy demanding cells. However, the understanding of how respiration is globally regulated is very limited. Interestingly, recent evidence suggests that Swi3 is an important regulator of respiration genes in yeast. In this report, we performed an array of biochemical and genetic experiments and computational analysis to directly evaluate the function of Swi3 and its human homologues in regulating respiration. First, we showed, by computational analysis and measurements of oxygen consumption and promoter activities, that Swi3, not Swi2, regulates genes encoding functions involved in respiration and oxygen consumption. Biochemical analysis showed that the levels of mitochondrial respiratory chain complexes were substantially increased in *Δswi3* cells, compared with the parent cells. Additionally, our data showed that Swi3 strongly affects haem/oxygen-dependent activation of respiration gene promoters whereas Swi2 affects only the basal, haem-independent activities of these promoters. We found that increased expression of aerobic expression genes is correlated with increased oxygen consumption and growth rates in *Δswi3* cells in air. Furthermore, using computational analysis and RNAi knockdown, we showed that the mammalian Swi3 BAF155 and BAF170 regulate respiration in HeLa cells. Together, these experimental and computational data demonstrated that Swi3 and its mammalian homologues are key regulators in regulating respiration.

## Abstract (precis)

Swi3 is a key component of the well-known SWI–SNF chromatin remodelling complex. Here, we discovered a novel Swi3 function: Swi3 and its mammalian homologues suppress oxygen consumption, and Swi3 regulates the expression of aerobic respiration genes in an oxygen-dependent manner.

## INTRODUCTION

Components of the SWI–SNF chromatin remodelling complex play essential roles in the development and proper functioning of a wide array of tissues and cell types, ranging from stem cells to neural and skin cells [1–4]. Their dysfunction is implicated in many cancers and neurological disorders. It is well known that Swi3 and its human homologues BAF155 and BAF170 are essential components of the SWI–SNF complexes, with Swi2 providing ATPase activity for chromatin remodelling [5–11]. The SWI–SNF complexes are found in virtually all eukaryotes [12]. Purified SWI–SNF complexes contain 10–12 polypeptides and have an apparent molecular mass of 1.14 MDa in yeast and approximately 2 MDa in mammals [5,6]. As part of this complex, Swi3 controls SWI–SNF assembly, ATP-dependent H2A–H2B displacement, as well as recruitment to target genes [7,8]. The target genes of SWI–SNF components constitute more than 10% of all yeast genes [13]. It is worth noting that both genetic data from yeast studies and chromatin immunoprecipitation sequencing (ChIP-Seq) data from mammalian cells showed that the target genes of SWI–SNF components do not completely overlap. Particularly, Swi3 and its mammalian homologues BAF155 and BAF170 can target a good number of genes and genomic locations in the absence of Swi2 and other components of the SWI–SNF complex [13,14], suggesting that they may have unique functions in gene regulation.

Furthermore, several lines of previous experimental evidence suggest that SWI–SNF proteins have important functions in oxygen regulation of gene expression. Firstly, by using fluorescent live cell imaging, we showed that six components of the SWI–SNF complex–Swi3, Snf5, Snf6, Snf11, Snf12 and Swp82–require oxygen for nuclear localization [15,16]. Under hypoxia, these proteins accumulate in the cytosol; upon reoxygenation, they relocalize to the nucleus. Notably, the changes in protein localization in response to hypoxia or reoxygenation precede the changes in transcriptome, showing a causal role of protein relocalization in promoting transcriptional changes [15]. Further, we characterized the time courses of relocalization of hypoxia-altered nuclear proteins in response to hypoxia and reoxygenation [16]. We found that Swi3, as well as 16 other nuclear proteins, responds to both hypoxia and reoxygenation in shorter times than the rest of the hypoxia-altered proteins. Secondly, we analysed mRNAs and proteins whose levels are regulated by oxygen levels, and found that many oxygen-regulated genes are targets of Swi3 and Swi2 [17]. Thirdly, Gat-Viks et al. [18] performed a gene sequence variation study identifying regulatory-linkage modules based on DNA sequence polymorphism and expression data. They showed that Swi3 is a dominant regulator in the control of respiratory gene expression; the effect of *swi3* deletion is stronger than that of known respiratory regulators, including Hap2/3/4/5, Mot3 and Rox1 [18]. All these results point out that Swi3 probably plays a unique role in oxygen regulation and the regulation of respiration.

Therefore, we decided to further ascertain the function of Swi3 in oxygen regulation and respiration. In this report, we confirmed that Swi3, but not Swi2, has a unique role in moderating respiration and oxygen consumption. Additionally, we showed that in cells with the *SWI3* gene deleted, the expression of genes encoding mitochondrial respiratory chain complexes is up-regulated. Further, we performed a computational analysis of genes bound by SWI–SNF proteins in mammalian cells, identified by the Snyder lab [14]. We found that the Swi3 homologues BAF155 and BAF170, but not the Swi2 homologue Brg1, are preferentially associated with genes encoding oxidative phosphorylation functions. We confirmed the role of BAF155 and BAF170 in respiration by measuring oxygen consumption in human HeLa cells with BAF155 or BAF170 knocked down. Together, these results uncover a unique role of Swi3 and its mammalian homologues in respiration and cellular bioenergetics.

## EXPERIMENTAL

### Yeast strains and plasmids

The yeast knockout and parent BY4741 (*MATa his3Δ1 leu2Δ 0 met15Δ0 ura3Δ0*) strains were purchased from Open Biosystems. The *HEM1* gene in the BY4741*Δhem1*, *Δswi3Δhem1*, *Δswi2Δhem1* strains were deleted as described previously [19]. To generate the BY4741*Δswi2Δswi3* strain, the BY4741* Δswi3* strain was transformed with PCR products containing the *LEU2* gene in the middle and 44 bps of sequences flanking the ORF sequence of *SWI2* on each end. The knockout strains were confirmed by using PCR analysis of the corresponding genomic DNA. Sequences for PCR primers are available upon request. The *CYC1-lacZ* and *CYC7-lacZ* reporters were described previously [20,21]. The *CYC1-lacZ* reporter contains -312 to -1 of the *CYC1* promoter region, whereas the *CYC7-lacZ* reporter contains -700 to -1 of the *CYC7* promoter region.

### Cell growth and β-galactosidase assays

Yeast cells were grown in rich YPD or synthetic complete media, as described previously [22,23]. Cell density was determined by measuring optical density at 600 nm. To determine β-galactosidase levels from reporter genes in *Δhem1* cells bearing the *CYC1-lacZ* or *CYC7-lacZ* reporter, cells were grown in synthetic complete medium containing a limiting amount of the haem precursor 5-aminolevulinate (2.5 μg/ml) or a high amount of 5-aminolevulinate (250 μg/ml). Cells were collected after they reached an optical density (600 nm) of approximately 1.0–1.5. Collected cells were then subjected to chloroform permeabilization β-galactosidase assays (in Miller units), as described previously [24].

### Cell lines, BAF155 and BAF170 knockdown, and Western blotting

HeLa cells were maintained in DMEM with 10% FBS under 5% CO_2_ at 37°C. To knockdown BAF155 and BAF170 in HeLa cells, target sequences were selected by using the computation program provided by Sigma–Aldrich. Then, shRNA pLKO.1-puro expression vectors and the corresponding Mission Lentiviral transduction particles were custom-made by Sigma–Aldrich. The vendor's recommended control particles were also used. HeLa cells were transduced with the viral particles, and puromycin-resistant clones were selected by following the vendor's procedures. Three knockdown clones were selected for BAF155 (629-1, 629-3, 630-2) and BAF170 (701-2, 701-3, 701-4) respectively. All cell culture reagents were purchased from Invitrogen. Puromycin was purchased from Sigma. The clones were confirmed by Western blotting.

For confirmation of knockdown by Western blotting, HeLa cells were collected and lysed by using the RIPA buffer (Cell Signaling Technology) containing the protease inhibitor cocktail. Protein concentrations were determined by using the bicinchoninic acid (BCA) assay kit (Thermo Scientific). Fifty micrograms of proteins were electrophoresed on 9% SDS/polyacrylamide gels, and then transferred on to the Immuno-Blot PVDF Membrane (Bio-Rad Laboratories). The membranes were probed with polyclonal antibodies, followed by detection with a chemiluminescence Western blotting kit (Roche Diagnostics). The signals were detected by using a Carestream image station 4000MM Pro. Polyclonal anti-BAF155 and anti-BAF170 were purchased from Santa Cruz Biotechnology. Monoclonal anti-β-actin antibody was purchased from Cell Signaling Technology.

### Measurement of oxygen consumption rate

Oxygen consumption was measured, as described previously [25]. HeLa cells were cultured to approximately 80% confluency, and were then trypsinized and resuspended in fresh, air-saturated medium. For each measurement, 10^6^ cells (in 350 μl) were introduced in the chamber of an Oxygraph system (Hansatech Instruments), with a Clark-type electrode placed at the bottom of the respiratory chamber. During measurements, the chamber was thermostated at 37°C by a circulating water bath. An electromagnetic stirrer bar was used to mix the contents of the chamber. For measuring oxygen consumption rate in yeast cells, 10^8^ cells (in 500 μl) were used, and the temperature was kept at 25°C.

### Analysis of mitochondrial respiratory chain complexes

Yeast parent BY4741, *Δswi2* and *Δswi3* deletion strains were grown in medium containing glucose or lactate (Lac) (note that *Δswi3* cells cannot grow in lactate medium) to mid-log phase. Cells were collected, and mitochondria were purified as described [26]. Purified mitochondria were solubilized and analysed on 4–12% acrylamide gradient Blue native PAGE gels, as described [27,28]. One hundred and fifty micrograms of total mitochondrial proteins was loaded in each lane.

### Computational analysis of Swi3, BAF155 and BAF170 target genes

The data for yeast Swi2 and Swi3 target genes were taken from Hu et al. [13]. Those genes identified as targets of Swi2 and Swi3 were used to perform the functional category enrichment analysis with DAVID (http://david.abcc.ncifcrf.gov/). All the KEGG (Kyoto Encyclopedia of Genes and Genomes) pathways and the GO (gene ontology) terms from biological processes (BP) and molecular functions (MF) were analysed. The ChIP-Seq data for BAF155, BAF170 and Brg1 targets are taken from Euskirchen et al. [14]. To identify the target genes of these proteins, we first identified genes whose promoters (defined as +/-2.5 kb of the transcription start site) are located in regions bound by these proteins. Then, we performed GO and KEGG pathway analysis by using the NIH DAVID program. The most significant GO groups and pathways with *P* values less than 0.05 were compared.

## RESULTS

### Computational analysis shows that Swi3 targets, but not Swi2 targets, are enriched in genes encoding functions involved in oxidation and reduction

Previously, Hu et al. [13] used gene expression data and directed-weighted graph modelling and regulatory epistasis analysis to characterize the regulatory network involving 263 transcription factors and to identify their target genes. These transcription factors include several components of the SWI–SNF complexes. Specifically, they identified 426 target genes for Swi2, with 63 unique to Swi2. Additionally, they identified 391 targets for Swi3, with 82 unique to Swi3. We performed GO and KEGG analyses of these unique targets and found that their function categories are completely different. Whereas Swi3 targets are enriched in metabolic enzymes, particularly those involved in oxidation and reduction, Swi2 targets are enriched in those involved in ribosome biogenesis and translation (see Table 1 for examples). No statistically significant GO groups of the Swi2 targets are relevant to oxidation and reduction, whereas no statistically significant GO groups of the Swi3 targets are relevant to ribosome. This analysis provides further support for investigating the unique function of Swi3 in regulating oxygen metabolism.

**Table 1:** The numbers of genes belonging to the top five GO classes of Swi2 and Swi3 only targets* *GO analysis was performed using NIH DAVID program. The five GO classes of genes with the lowest *P* values are shown here. The values in the parentheses are *P* values.

	Total	Response to temperature stimulus	Vacuolar protein catabolic process	Response to abiotic stimulus	Oxidation reduction	Oxidative phosphorylation
Swi3	82	11 (0.0003)	8 (0.0006)	13 (0.001)	12 (0.004)	5 (0.006)
	**Total**	**Ribosome**	**Translation**	**rRNA metabolic process**	**Maturation of SSU-rRNA**	**RNA binding**
Swi2	63	19 (8.3E-14)	28 (3.6E-10)	11 (0.0004)	7 (0.0004)	16 (0.0007)

### Swi3, but not Swi2, strongly affects oxygen consumption and respiration

**Figure 1:**
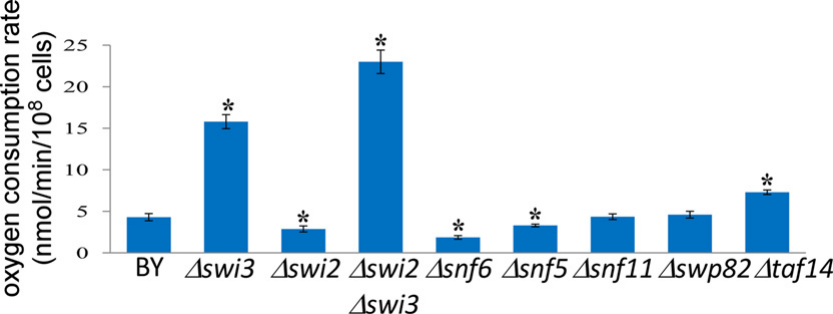
The effect of deleting the genes encoding components of the SWI–SNF complex on oxygen consumption rate Yeast parent BY4741 (BY) and knockout strains were grown to mid-log phase and collected for measuring oxygen consumption. The data shown are averages of at least three independent measurements. For statistical analysis, the values were compared with those in the parent strain (BY), by using Welch two-sample *t* test. *, *P* value<0.005.

**Figure 2:**
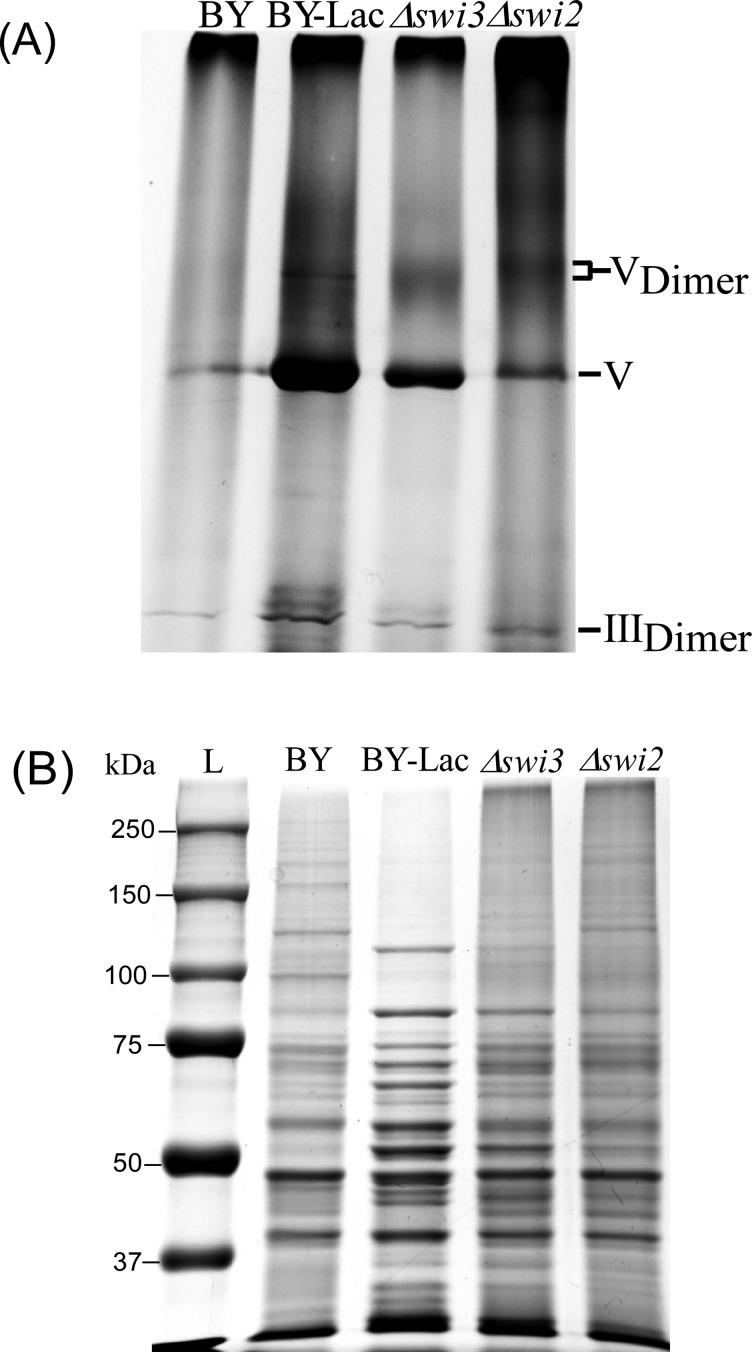
The effect of deleting the *SWI3* gene on the expression of mitochondrial respiratory chain complexes Yeast parent BY4741 (BY), *Δswi2* and *Δswi3* deletion strains were grown in medium containing glucose or lactate (Lac) (note that *Δswi2* and *Δswi3* cells cannot grow in lactate medium) to mid-log phase. Cells were collected, and mitochondria were purified. Purified mitochondria were solubilized and analysed on 4–12% acrylamide gradient BN gels (**A**). In (**B**), the same samples were analysed by using SDS/PAGE.

To ascertain the function of Swi3 and other SWI–SNF components in respiration, we measured the rates of oxygen consumption in various deletion strains and compared them with that of the parent strain BY4741 (Figure 1). Evidently, the rate of oxygen consumption was significantly elevated in *Δswi3* cells, compared with the parent BY4741 cells. However, the rate of oxygen consumption was not enhanced in *Δswi2* cells or the cells with one of the other SWI–SNF genes deleted, except for *Δtaf14* cells (Figure 1). Notably, deletion of the SWI2 gene in *Δswi3* cells did not diminish intensified oxygen consumption, suggesting that Swi3 acts independently of Swi2. Further, we measured the levels of mitochondrial respiratory chain complexes in the parent and *Δswi3* cells. We prepared purified mitochondria and analysed them by blue native (BN) gel electrophoresis [28,29] (Figure 2). We found that increased oxygen consumption in *Δswi3* cells is correlated with increased levels of mitochondrial respiratory chain complexes. Because lactate is a non-fermentable carbon source and cells grown in lactate should express high levels of oxidative phosphorylation complexes [20], we analysed the complexes from the parent cells grown in lactate for a positive control and for comparison. As expected, the levels of the complexes isolated from the parent cells grown in lactate (see V_Dimer_, V, and III_Dimer_, Figure 2A, BY lac) were much higher than those from the parent (BY, Figure 2A) or *Δswi2* cells grown in glucose. Although BN gel electrophoresis can detect clearly oxidative phosphorylation complexes formed in mammalian cells, it is often not sensitive enough to detect all complexes formed in yeast, as shown in previous work [30]. Notably, the level of the complexes in *Δswi3* cells grown in glucose was significantly higher than that in parent cells grown in glucose (BY). The level of the complexes was somewhat increased in *Δswi2* cells, but lower than that in *Δswi3* cells. The same samples were also analysed by using SDS/PAGE (Figure 2B), in order to show that the amounts of total proteins in these samples were similar. This result showing increased level of oxidative phosphorylation complexes is consistent with enhanced oxygen consumption, in *Δswi3* cells.

### Swi3, but not Swi2, is critical for haem-dependent activation of genes encoding functions involved in respiration

**Figure 3:**
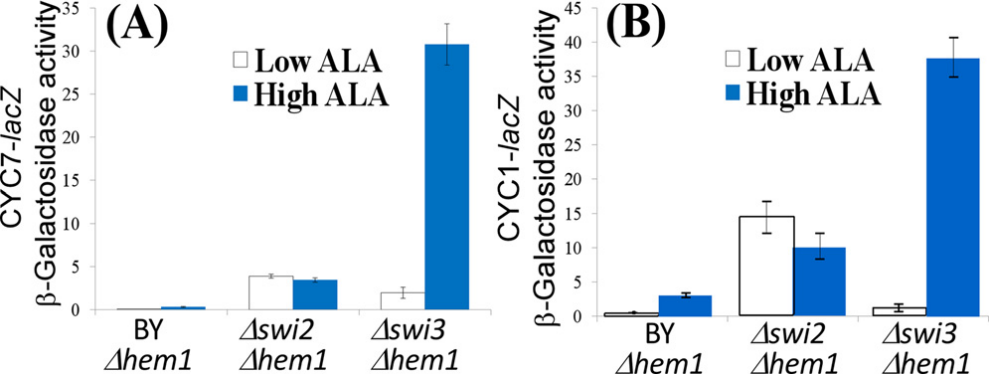
Swi2 and Swi3 exert differential effects on respiration gene transcription Deletion of the SWI3 gene, not SWI2, causes a significant increase in the *CYC7* (**A**) and *CYC1* (**B**) promoter reporter activities in haem-sufficient cells. Yeast parent BY4741*Δhem1* (BY*Δhem1*), BY4741*Δswi2Δhem1* (*Δswi2Δhem1*) and BY4741*Δswi3Δhem1* (*Δswi3Δhem1*) strains bearing the *CYC1-lacZ* or *CYC7-lacZ* reporter plasmid were grown in the presence of 2.5 μg/ml (low ALA) or 250 μg/ml (high ALA) haem precursor ALA. β-Galactosidase activities were measured and plotted here. Data shown here are averages from at least three independent measurements.

To further probe the mechanism by which Swi3 affects respiration, we examined the effect of deleting *SWI2* or *SWI3* on the promoter activities of two previously characterized respiration genes *CYC1* and *CYC7*, encoding cytochrome *c* iso-1 and iso-2 respectively [20,21,31]. We measured the β-galactosidase activities of the full *CYC1* promoter-*lacZ* and *CYC7* promoter-*lacZ* reporters in *Δswi2* and *Δswi3* cells, under haem-sufficient and haem-deficient conditions (Figure 3). This assay measuring promoter activities is much more sensitive in detecting changes in respiration gene expression. Note that intracellular haem levels can be controlled by deleting the *HEM1* gene and adding back different levels of the haem precursor 5-aminolevulinic acid (ALA). Additionally, the level of haem synthesis is tightly controlled by oxygen level [22]. Haem is a cofactor for mitochondrial respiratory chain complexes II, III and IV [32], and it promotes the expression of respiration genes. Thus, altering intracellular haem levels provides a convenient way to modulate respiration. In the BY4741 strain, the haem-activated transcriptional activator Hap1, which promotes the expression of respiration genes under aerobic conditions, lacks the activation domain and is defective in transcriptional activation [33]. Thus, the promoter activities of respiration genes are low in the parent strain even in haem-sufficient cells, as shown in Figure 3. In haem-deficient *Δswi2* cells, the activities of the reporters were significantly higher than those in haem-deficient parent cells (Figure 3). High haem levels in haem-sufficient *Δswi2* cells did not cause a further increase in reporter activities. This suggests that Swi2 generally affects the promoter activities in a haem-independent manner. In haem-deficient *Δswi3* cells, the activities of the reporters were higher than those in haem-deficient parent cells, but were not as high as those in haem-deficient *Δswi2* cells (Figure 3). High haem levels in haem-sufficient *Δswi3* cells further caused a substantial increase in reporter activities. This suggests that Swi3 strongly affects haem/oxygen-dependent promoter activities but exerts a weaker haem-independent effect on the promoters than Swi2.

### Increased activation of respiration gene expression correlates with intensified oxygen consumption and cell growth in *Δswi3* cells

**Figure 4:**
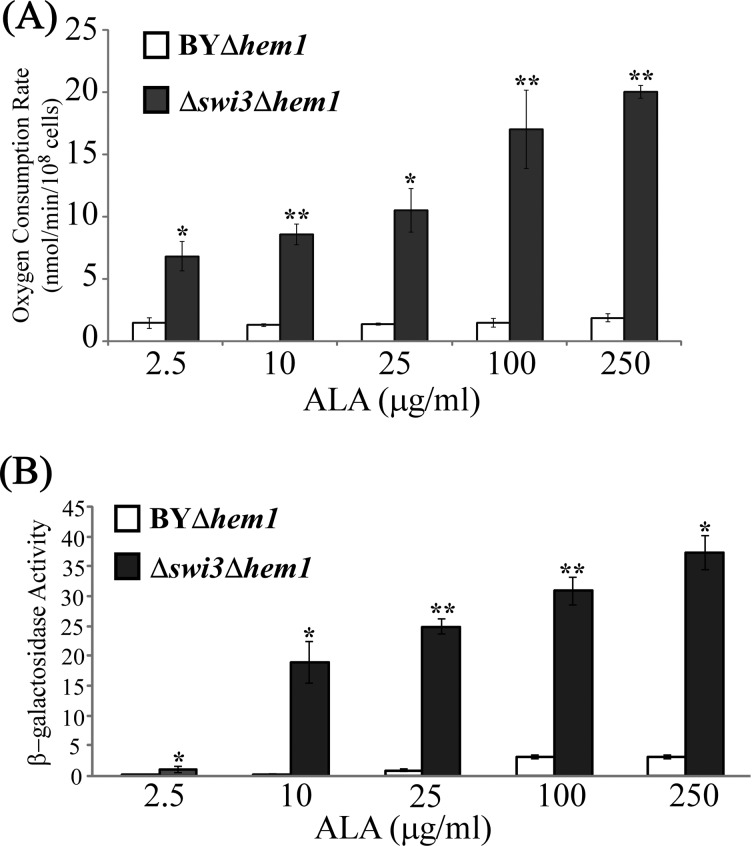
Haem promotes the rates of oxygen consumption (**A**) and CYC7 promoter activity (**B**) in *Δswi3* cells Yeast parent BY4741*Δhem1* (BY*Δhem1*) and BY4741*Δswi3Δhem1* (*Δswi3Δhem1*) cells bearing the *CYC7-lacZ* reporter were grown in the presence of indicated levels of haem precursor ALA. Cells were grown to mid-log phase and collected for measuring oxygen consumption or β-galactosidase activities. The data shown are averages of at least three independent measurements. For statistical analysis, the values were compared with those in the parent strain (BY), by using Welch two-sample *t* test. *, *P* value < 0.005; **, *P* value < 0.001.

**Figure 5:**
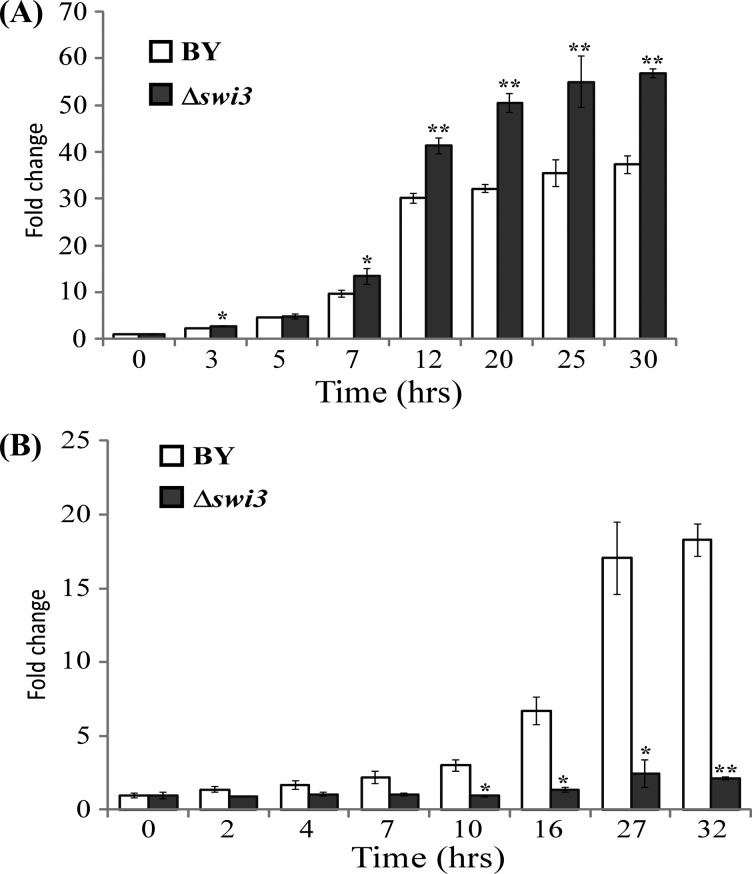
Deletion of the *SWI3* gene selectively enhances cell growth rate in air Yeast parent BY4741 (BY) and BY4741*Δswi3* (*Δswi3*) were grown in air (**A**) or under hypoxia (**B**). Cells were grown to mid-log phase and collected for measuring optical density. Fold change was calculated by dividing the optical density at the indicated time points with that at the designated 0 h time. The data shown are averages of at least three independent measurements. For statistical analysis, the values were compared with those in the parent strain (BY), by using Welch two-sample *t* test. *, *P* value < 0.005; **, *P* value < 0.001.

To further ascertain the association of haem and respiration gene expression with oxygen consumption in *Δswi3* cells, we measured the rates of oxygen consumption in the parent BY4741*Δhem1* and *Δswi3Δhem1* cells and compared them to *CYC7* promoter activities, under increasing concentrations of the haem precursor ALA. Figure 4 shows that as haem levels increased, the rate of oxygen consumption, along with the CYC7 reporter activity, increased substantially in *Δswi3Δhem1* cells, but it did not increase in BY4741*Δhem1* cells. Further, we found that in air, the rate of *Δswi3* cell growth was increased compared with the parent cells (Figure 5A). However, under hypoxia, in the absence of respiration, *Δswi3* cell growth rate was much lower than the parent cells (Figure 5B). These results together show that the loss of Swi3 function promotes the expression of respiration genes and the formation of mitochondrial respiratory chain complexes, leading to increased oxygen consumption and cell growth in air. Additionally, the data suggest that the function of Swi3 in aerobic cells is distinct from its function in hypoxic cells.

### Computational analyses of genome-wide ChIP-Seq data about BAF proteins suggest that the human Swi3 homologues BAF155 and BAF170 are important for respiration gene expression

The functions of SWI–SNF proteins are highly conserved from yeast to humans. Particularly, Swi3 has two homologues in humans, namely BAF155 and BAF170. These three proteins contain three conserved functional domains, namely SWIRM, SANT and LZ domains [34,35]. To comprehensively survey the genome-wide localization of SWI–SNF proteins, three ChIP-Seq studies have been performed in ES cells, T helper cells and HeLa cells [14,36,37]. In particular, the Snyder lab detected and compared the localization patterns of Brg1, BAF155, BAF170 and Ini1 [14]. Analysis of their data (see Table 2) showed that BAF155 and BAF170 localize to many regions/genes where Brg1 does not, suggesting that BAF155 and BAF170 have other functions besides those requiring Brg1.

**Table 2:** Pathway analysis of genes bound by BAF155, BAF170 and/or Brg1* *Shown here are the total number (Total) of genomic regions associated with BAF155 and BAF170, but not Brg1 ([155+170]-Brg1); with BAF155, BAF170 and Brg1 ([155+170+Brg1]); with only BAF155 (155 only); with only BAF170 (170 only) and with only Brg1 (Brg1 only). These regions that are within promoters of genes were identified, and the number of genes (gene promoters) are shown. KEGG pathway analysis was then performed with these groups of genes by using the NIH DAVID program. Three most significant pathways for each group of genes are shown here. The numbers in parentheses indicate the number of genes in the pathway and the corresponding *P* value. OxP: oxidative phosphorylation; CML: chronic myeloid leukaemia; Ins: insulin signalling pathway; Cancer: pathways in cancer; p53: p53 signalling pathway; Ad junc: adherens junction; HCM: hypertrophic cardiomyopathy; ARVC: arrhythmogenic right ventricular cardiomyopathy; Melano: melanogenesis; Endo: endocytosis; Hedge: Hedgehog signalling pathway. No known genes were found to be in regions that are bound by only Brg1.

	[155+170]-Brg1	[155+170+Brg1]	155 only	170 only	Brg1 only
Total	13198	10005	8381	1590	281
Gene promoters	4891	3737	1540	321	40
	OxP (47, 4.7E-5)	Cancer (86, 6.5E-6)	Ad junc (15, 4.0E-4)	Melano (6, 3.0E-2)	None
KEGG	CML (32, 2.8E-5)	CML (25, 7.2E-4)	HCM (13, 9.0E-3)	Endo (8, 4.3E-2)	
(Genes, *P* value)	Ins (48, 6.3E-5)	p53 (24, 3.8E-4)	ARVC (13, 1.0E-2)	Hedge (4, 7.6E-2)	

To probe whether the role of Swi3 in respiration is conserved in mammalian cells, we analysed and compared the regions bound by BAF155, BAF170 and Brg1, using ChIP-Seq data from Euskirchen et al. [14] (Table 2). First, we identified genes whose promoters (defined as +/-2.5 kb of the transcription start site) are located in regions bound by these proteins (Gene Promoters in Table 1). Then, we performed GO (gene ontology) and KEGG (Kyoto Encyclopedia of Genes and Genomes) pathway analysis. Strikingly, the analysis (Table 2) showed that among the genes bound by both BAF155 and BAF170, but not by Brg1, a significant number [47] of them encode enzyme subunits of the oxidative phosphorylation (mitochondrial respiratory chain) pathway. In contrast, among the genes bound by BAF155, BAF170 and Brg1, those associated with oxidative phosphorylation were not present. No statistically significant GO groups of the Brg1 targets are relevant to oxidative phosphorylation. The results suggest that oxidative phosphorylation/respiration genes are modulated by both BAF155 and BAF170, but this modulation does not require Brg1.

### RNAi knockdown of BAF proteins shows that BAF155 and BAF170 affect oxygen consumption in mammalian cells

**Figure 6:**
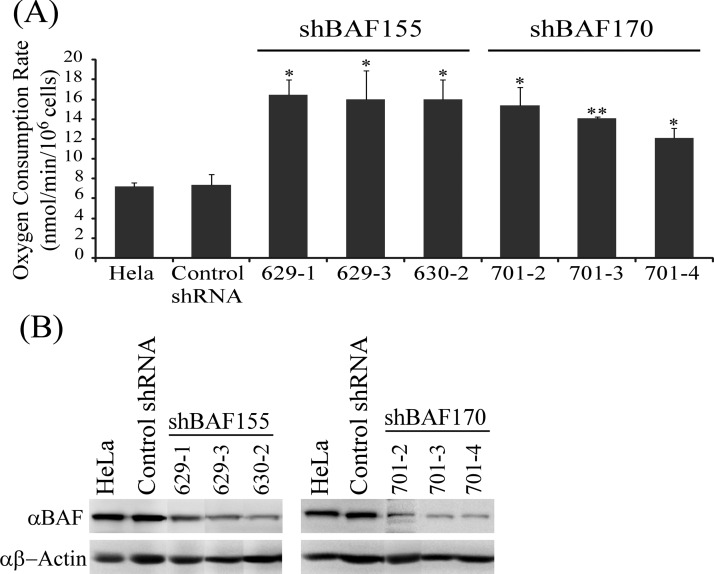
The effect of knocking down BAF155 and BAF170 on the rate of oxygen consumption in HeLa cells (**A**) The rates of oxygen consumption in HeLa clones with BAF155 or BAF170 knocked down. To measure oxygen consumption rate, 10^6^ cells (in 350 μl) of each clone were introduced in the chamber of an Oxygraph system (Hansatech Instruments). The values plotted are averages from at least three experiments. For statistical analysis, the levels in HeLa clones were compared with the levels in the control clone, by using Welch two-sample *t* test. *, *P* value < 0.05; **, *P* value < 0.005. (**B**) Western blot showing the levels of BAF155 and BAF170 in HeLa cell clones. HeLa cells were transduced with Mission Lentiviral transduction particles bearing shRNA pLKO.1-puro expression vectors for targeting BAF155 (629-1, 629-3 and 630-1) or BAF170 (701-2, 701-3 and 701-4) or for control (does not target any mammalian genes) (Sigma–Aldrich). Puromycin-resistant clones were selected by following the vendor's procedures. Protein extracts were prepared from the positive clones and analysed by using Western blotting.

To further ascertain the role of BAF155 and BAF170 in regulating respiration, we knocked down BAF155 and BAF170 and examined the effect on oxygen consumption (Figures 6A and 6B). We selected HeLa cell clones that stably expressed target shRNAs and exhibited reduced levels of BAF155 or BAF170 (Figure 6B). In those clones, the rates of oxygen consumption were significantly increased compared with that in control cells (Figure 6A). These results show that both BAF155 and BAF170, acting independently or cooperatively, modulate oxygen consumption.

## DISCUSSION

The dysregulation of cellular bioenergetics has increasingly been found to be associated with a wide array of human diseases, including cancer, neurological diseases and diabetes [1,38–40]. Given that oxidative phosphorylation/mitochondrial respiration yields 18 times more ATP per glucose molecule compared with glycolysis, it is conceivable that respiration is germane to the regulation of cellular bioenergetics in many eukaryotic cells, particularly cells that require a high level of energy supply. One kind of such cells are brain cells. The brain uses 20% of oxygen consumed by the whole human body, even though its mass is only 2% of the whole body. Another kind of such high energy demanding cells are cancer cells. Therefore, understanding the molecular mechanisms governing the regulation of cellular bioenergetics is of fundamental importance to the prevention and treatment of many common diseases.

In this report, we present a new series of experiments to directly ascertain the function of Swi3 and its human homologues in the control of respiration, based on previous indirect experimental and computational evidence suggesting a link between Swi3 and oxygen regulation and aerobic gene expression [15–18]. First, by analysing previously identified targets of SWI–SNF proteins, we showed that Swi2 and Swi3 have functions separate from other SWI–SNF components and that Swi3, not Swi2, has a tendency to target genes encoding functions involved in oxidation and reduction or oxidative phosphorylation (Table 1). Next, we showed that Swi3 has a unique function in moderating oxygen consumption in wild-type cells, because deletion of the *SWI3* gene, not the *SWI2* gene, caused a substantial increase in oxygen consumption rate (Figure 1). Biochemical analysis showed that the levels of mitochondrial respiratory chain complexes were substantially increased in *Δswi3* cells, compared with the parent cells (Figure 2). Next, we measured the promoter activities of two representative genes (*CYC1* and *CYC7*) encoding functions involved in mitochondrial respiration in haem-sufficient and haem-deficient cells. Our results showed that Swi3 strongly affects haem/oxygen-dependent activation of *CYC1* and *CYC7* promoters whereas Swi2 affects only the basal, haem-independent activities of these promoters (Figure 3). Measurement of oxygen consumption rates and cell growth rates (Figures 4 and 5) showed that increased expression of respiration genes is correlated with increased oxygen consumption rate and growth rate in *Δswi3* cells in air.

Further, we ascertained that Swi3 homologues in mammalian cells play a role in moderating respiration. We first identified the unique targets of the Swi3 homologues BAF155 and BAF170, based on the genome-wide ChIP-Seq data from Euskirchen et al. [14] (Table 2). We then analysed these targets and found that BAF155 and BAF170 localize to many oxidative phosphorylation/respiration genes whereas the Swi2 homologue Brg1 does not. Subsequently, we confirmed the roles of BAF155 and BAF170 in moderating oxygen consumption by detecting the oxygen consumption rate in HeLa cells with BAF155 or BAF170 knocked down (Figure 6). Together, these experimental and computational data demonstrated that Swi3 and its mammalian homologues is a master regulator in moderating oxygen consumption and oxidative phosphorylation.

Our results provide a novel mechanism for the action of Swi3 human homologues, and perhaps homologues of other SWI–SNF proteins in the pathogenesis of cancer and neurological diseases. The mutations of Swi3 and other SWI–SNF proteins have been shown to be associated with a range of cancers [38,39]. Respiration has been increasingly shown to provide critical cellular energy supply in many types of cancer cells [41–45]. Swi3 moderates oxygen consumption and respiration, thereby limiting cellular energy generation and promoting controlled cellular function. Loss of function of Swi3 would lead to intensified oxygen consumption and cellular energy production, thereby promoting tumorigenic transformation of cellular functions.

Swi3 homologues and other SWI–SNF proteins have been suggested to play important roles in the nervous systems [1,46]. Neuronal cells have a high number of mitochondria and continuously require a high amount of cellular energy from respiration. Indeed, several lines of experimental evidence have shown that the Swi3 mammalian homologues BAF155 and BAF170 are critical for brain development and brain function. For example, Kim et al. [47] showed that BAF155 is essential for early embryogenesis and plays an important role in brain development in mice. Recently, Tuoc et al. [48,49] showed that BAF170 is an intrinsic factor that controls cortical size and thickness in mice. It has been presumed that this function of BAF155 and BAF170 is attributable to their involvement in the mSWI–SNF complexes, because this has been the well-known biochemical property of these proteins. However, our new finding demonstrating a role of Swi3, BAF155 and BAF170 in oxidative phosphorylation provides a new, likely mechanism for these proteins to affect neuronal functions and brain development. If BAF155 and BAF170 indeed modulate cellular bioenergetics in a variety of eukaryotic cells, their dysfunction may underlie the pathogenesis of a wide range of common diseases, including cancer, neurological diseases and diabetes.

## Abbreviations

BN: blue native
ChIP-Seq: chromatin immunoprecipitation sequencing
